# Paeoniflorin exerts neuroprotective effects by modulating the M1/M2 subset polarization of microglia/macrophages in the hippocampal CA1 region of vascular dementia rats via cannabinoid receptor 2

**DOI:** 10.1186/s13020-018-0173-1

**Published:** 2018-03-20

**Authors:** Xian-Qin Luo, Ao Li, Xue Yang, Xiao Xiao, Rong Hu, Tian-Wen Wang, Xiao-Yun Dou, Da-Jian Yang, Zhi Dong

**Affiliations:** 1 Chongqing Key Laboratory of Biochemistry and Molecular Pharmacology, School of Pharmacy, Chongqing Medical University, Chongqing, 400016 China; 2 College of Pharmacy and Bioengineering, Chongqing University of Technology, Chongqing, 400054 China; 3 Institute of Chinese Pharmacology and Toxicology, Chongqing Academy of Chinese Materia Medica, Chongqing, 400065 China; 4 Drug Review Section, China Chongqing Technical Center for Drug Evaluation and Certification, Chongqing, 400014 China; 5 Institute of Life Sciences, Chongqing Medical University, Chongqing, 400016 China

**Keywords:** Paeoniflorin, Cannabinoid receptor 2, Vascular dementia, Neuroprotection

## Abstract

**Background:**

Cerebral hypoperfusion is a pivotal risk factor for vascular dementia (VD), for which effective therapy remains inadequate. Persistent inflammatory responses and excessive chemotaxis of microglia/macrophages in the brain may accelerate the progression of VD. Endocannabinoids are involved in neuronal protection against inflammation-induced neuronal injury. Cannabinoids acting at cannabinoid receptor 2 (CB_2_R) can decrease inflammation. Based on the identification of paeoniflorin (PF) as a CB_2_R agonist, we investigated the neuroprotective and microglia/macrophages M1 to M2 polarization promoting effects of PF in a permanent four-vessel occlusion rat model.

**Methods:**

One week after surgery, PF was intraperitoneally administered at a dose of 40 mg/kg once a day for 28 successive days. The effects of PF on memory deficit were investigated by a Morris water maze test, and the effects of PF on hippocampal neuronal damage were evaluated by light microscope and electron microscope. The mRNA and protein expression levels of key molecules related to the M1/M2 polarization of microglia/macrophages were assessed by RT-qPCR and Western blotting, respectively.

**Results:**

Administration of PF could significantly attenuate cerebral hypoperfusion-induced impairment of learning and memory and reduce the morphological and ultrastructural changes in the hippocampal CA1 region of rats. Moreover, PF promoted an M1 to M2 phenotype transition in microglia/macrophages in the hippocampus of rats. In addition to its inhibitory property against proinflammatory M1 mediator expression, such as IL-1β, IL-6, TNF-α and NO, PF dramatically up-regulated expression of anti-inflammatory cytokines IL-10 and TGF-β1. Importantly, CB_2_R antagonist AM630 abolished these beneficial effects produced by PF on learning, memory and hippocampus structure in rats, as well as the polarization of microglia/macrophages to the M2 phenotype. Additionally, PF treatment significantly inhibited cerebral hypoperfusion-induced mTOR/NF-κB proinflammatory pathway and enhanced PI3K/Akt anti-inflammatory pathway. Effects of PF on these signaling pathways were effectively attenuated when rats were co-treated with PF and AM630, indicating that the mTOR/NF-κB and PI3K/Akt signaling pathways were involved in the PF effects through CB_2_R activation.

**Conclusion:**

These findings demonstrated PF exerts its neuroprotective effect and shifts the inflammatory milieu toward resolution by modulation of microglia/macrophage polarization via CB_2_R activation.

**Electronic supplementary material:**

The online version of this article (10.1186/s13020-018-0173-1) contains supplementary material, which is available to authorized users.

## Background

Vascular dementia (VD) is a cognitive impairment syndrome caused by cerebral hemorrhage, ischemia or acute/chronic cerebral hypoxia, and other cerebrovascular diseases. VD often presents as an impairment in memory, speech, calculation, and visuospatial functions, and severely affects the patient’s daily life [1, 2]. Dementia due to VD accounts for 20% of all cases of dementia, with an incidence secondary only to dementia caused by Alzheimer’s disease [3]. With an increasing human lifespan and an increase in the incidence of cerebrovascular diseases, the population of people affected by VD is increasing year-by-year, leading to huge burdens on families and society [2].

The pathogenesis of VD is still not completely clear. Neurological imaging and pathological studies have shown that pathological changes due to cerebrovascular obstruction or injury results in insufficient cerebral perfusion and are the main cause of VD-induced cognitive impairments [4]. In addition, inflammatory responses are an important factor of secondary brain injury caused by cerebral ischemia. Studies have found that after ischemic brain damage, microglial cells in the brain and macrophages that infiltrated the central nervous system (CNS) due to damage to the blood–brain barrier will rapidly migrate to the site of inflammation. In the damaged environment, these cells are activated through the classical pathway to undergo M1 polarization and secrete large amounts of reactive oxygen species and inflammatory mediators, such as IL-1β, tumor necrosis factor-α (TNF-α), and nitric oxide (NO), which promote inflammatory responses in the CNS and accelerate neuronal death. In the later stage of neuroinflammation, microglia/macrophages that are polarized into the M2 phenotype by the alternative activation pathway can secrete arginase-1, IL-10, and other anti-inflammatory factors. This will inhibit synaptic damage due to excessive inflammation and promote the regeneration of axons. Therefore, regulating the activation status of microglia/macrophages can be a new treatment strategy for VD.

In the CNS, neurotransmission and neuroinflammation are mediated by the cannabinoid signaling system. Cannabinoid receptors include cannabinoid receptor 1 (CB_1_R) and cannabinoid receptor 2 (CB_2_R). CB_1_R is primarily expressed in the CNS and peripheral neuronal tissues and mainly regulates neuropsychological functions, while CB_2_R belongs to the Gi/Go G-protein-coupled receptor. CB_2_R is mainly expressed in cells associated with innate immunity, such as microglial cells, dendritic cells, and cerebrovascular endothelial cells, and participates in immunoregulation in the brain. In VD animal models, selective agonists of CB_2_R can decrease memory impairments and infarction areas in rats caused by insufficient cerebral perfusion and vascular dementia [5]. Similarly, for microglial cells and neurons that are cultured in vitro, endocannabinoids or the CB_2_R agonist JWH-015 can decrease lipopolysaccharide (LPS)-induced synthesis of IL-1β, TNF-α and NO [6, 7]. In cultures of mouse cortical microglial cells, activated CB_2_R can induce the synthesis of the anti-inflammatory cytokine IL-10, inhibiting the activation of microglial cells and suppressing neuroinflammation [8]. These study results mentioned above suggest that CB_2_R activation can aid in converting M1 microglia/macrophages during the recovery phase of cerebral ischemia into the M2 phenotype, thereby decreasing inflammatory responses after cerebral ischemia and promoting the recovery of neurobehavioral functions.

Besides IFN-γ, IL-4, and other classical induction factors, more regulatory factors that induce conversion of microglia/macrophage polarization have been identified recently. As an example, it has been shown that increasing mammalian target of rapamycin (mTOR) activity could significantly increase the survival of microglial cells in an in vitro ischemia/hypoxia model that is deprived of oxygen and glucose. In addition, inducible nitric oxide synthase (iNOS) expression was induced, and this promoted NO synthesis [9]. In contrast, inhibition of mTOR activity can significantly decrease microglia activation in the glucose and oxygen deprivation model, inducing its conversion from the M1 phenotype to an M2 phenotype [10]. At the same time, this ameliorates learning and memory impairments in mice caused by insufficient cerebral perfusion. Furthermore, in microglial cells that have been transfected with interferon regulatory factor 3-overexpressing gene via adenoviruses, up-regulation of the phosphatidylinositol-3-kinase (PI3K)/protein kinase B (PKB/Akt) signaling pathway can similarly mediate the conversion of microglial cells from an M1 phenotype into an M2 phenotype [11]. In mouse microglial cells, β-amyloid can activate nuclear factor-κB (NF-κB) signaling and at the same time reduce PI3K/Akt signaling, leading to down-regulation of mRNA expression of M2 phenotype marker genes [12]. In summary, the results presented above suggest that the mTOR, NF-κB and PI3K/Akt signaling pathways may participate in the conversion of the M1/M2 polarization of microglia/macrophages after CB_2_R activation.

Previously, we have established a CB_2_R agonist screening system using a combination of PIRES2-EGFP-CB_2_R eukaryotic expression plasmid and the luc2P/CRE/Hygro reporter gene [13, 14]. Using this system, we screened more than 100 ingredients and extracts used in the traditional Chinese medicine and found that paeoniflorin (PF) shows the strongest CB_2_R stimulation activity. PF, a monoterpene glycoside, is one of the main active ingredients of the traditional Chinese medicine *Paeonia lactiflora* Pall and *Paeonia veitchii* Lynch. Studies in recent years have found that PF can activate the muscarinic acetylcholine receptors [15] and adrenaline receptors [16] to improve spatial recognition as well as learning and memory functions. PF can also elicit analgesic effects by acting on opioid receptors [17]. Several studies have implicated that PF acting through adenosine A_1_ receptors decreases infarction caused by acute or chronic cerebral ischemia and alleviates neurobehavioral impairments [18]. Recent studies have also found that PF can significantly improve cognitive impairments in VD rat models by increasing cerebral blood flow and inhibiting the hippocampal neuronal apoptosis and NF-κB-mediated inflammatory signaling pathway [19, 20]. Our previous data also showed that PF could protect hippocampal neurons in rats subjected to cerebral ischemia–reperfusion through activating CB_2_R [21]. However, there are no reports so far on whether PF can activate CB_2_R to inhibit cerebral ischemia-induced polarization of microglia/macrophages in the hippocampal regions and thus protect adjacent neurons and restore spatial memory and cognitive functions. Therefore, we studied here a four-vessel occlusion (4-VO) rat model to delineate the potential molecular mechanisms by which PF inhibits neuroinflammation and elicits its neuroprotective effects in a CB_2_R dependent manner.

## Methods

### Information of experimental design and resources

The Minimum Standards of Reporting Checklist (Additional file 1) contains details of the experimental design, and statistics, and resources used in this study.

### Reagents

Paeoniflorin (> 98% purity) was obtained from Shanghai Daibo Chemical Technology Co., Ltd (Shanghai, China); HU308 (a selective CB_2_R agonist) and AM630 (a selective CB_2_R antagonist) were obtained from Cayman Chemicals (Ann Arbor, MI, USA). CB_2_R antibody was obtained from Thermo Fisher Scientific Inc. (Waltham, MA, USA), NF-κB p65 antibody was obtained from Abcam Inc (Cambridge, MA, UK), Phospho-Akt (Ser473), Akt, mTOR, Phospho-mTOR (Ser2448) and Phospho-IκBα (Ser32) antibodies were obtained from Cell Signaling Technology Inc. (Beverly, MA, USA), iNOS, CD206, IκBα and lamin B1 antibodies were obtained from Santa Cruz Biotechnology Inc. (Santa Cruz, CA, USA), CD68 antibody was obtained from AbD Serotec (Kidlington, Oxford, UK), Iba1 was from Wako Pure Chemical (Osaka, Tokyo, Japan), and phospho-PI3K p85α (Tyr467)/phospho-PI3K p55γ (Tyr199), PI3K and β-actin antibodies were obtained from Bioworld Technology Inc. (Minneapolis, MN, USA). An NO assay kit was obtained from Beyotime Institute of Biotechnology (Jiangsu, China).

### Animals

Male Sprague–Dawley (SD) rats, weighing 300‒400 g, SPF grade, were obtained from the Experimental Animal Research Center of Chongqing Academy of Chinese Materia Medica; Approval No. SCXK (Yu) 2012-0006. All rats were housed in the experimental animal room of the Chongqing Academy of Chinese Materia Medica with free access to food and water, under a 12:12 h light–dark cycle, at a constant environmental temperature of 22‒25 °C and a relative humidity of 50‒60%. The animals were allowed to acclimatize for 1 week before the experiments. All protocols in this study conformed to the National Institutes of Health guidelines for the Care and Use of Laboratory Animals (8th Edition, 2011), and the use of animals was approved by the animal ethics committee of the Chongqing Academy of Chinese Materia Medica.

### Surgery

Rats were subjected to the Morris water maze test, and rats that failed to find the platform within 90 s were excluded. Four-vessel occlusion was carried out in qualified rats according to the method described by Pulsinelli et al. [22, 23]. After the rats were fasted for 24 h, intraperitoneal injections of 45 mg/kg pentobarbital sodium were used to anesthetize the rats. The rats were fixed on the operating station in a prone position and routine skin preparation was carried out. An incision was made in the middle of the neck, and the latissimus dorsi and trapezius muscles were gradually separated. The transverse foramen on the left and right sides of the 1st cervical vertebra was exposed and a 0.5-mm diameter electrocoagulation needle was inserted 1‒2 mm into the transverse foramen from the tail end, to cause permanent occlusion of the vertebral arteries. Twenty-four hours later, the rats were re-anesthetized by intraperitoneal injections of 40 mg/kg pentobarbital sodium. The rats were kept at the supine position, and an incision was made in the middle of the neck. Blunt separation was carried out on the common carotid arteries on both sides, and non-invasive microvascular clips were used to clip both common carotid arteries to induce cerebral ischemia. Ten minutes later, the vascular clips were removed to restore the blood flow to the carotid arteries. In order to decrease inter-animal differences, the following conditions had to be strictly met during the 10-min ischemia period and the subsequent 20 ± 5-min comatose stage: loss of righting reflex and dilation of both pupils. During the 6 h after ischemia, the anal temperature of the rats was maintained at 37 ± 0.5 °C. Besides no vertebral artery cauterization on the first day and ischemic treatment of bilateral common carotid arteries on the second day in the sham treatment group, the remaining procedures were identical to those performed in the treatment group.

### Animal grouping and drug treatment

One week after surgery, 120 rats of dementia were selected, based on the results from the Morris water maze with the following success criterion: using the mean value of the time taken by sham-operated rats to escape from the maze as a reference value, rats with a ratio of the difference between the escape latency period and the reference value greater than 20% were defined as dementia rats [24, 25]. In addition to the sham-operated group (*n *= 24), successful models were randomly assigned to five groups (*n *= 24): the model group, the PF (40 mg/kg) group (Additional file 2: Figure S1), the PF (40 mg/kg) + AM630 (3 mg/kg) group, the AM630 (3 mg/kg) group, and the HU308 (3 mg/kg) group. All drugs were dissolved in Tween 80 and dimethyl sulfoxide (DMSO), and then was diluted by saline (Tween 80:DMSO:saline = 1:1:18), which was administered intraperitoneally to rats at a volume of 5 ml/kg 15 min after permanent four-vessel occlusion and then with the same dose once a day for 27 successive days. In order to study the role of CB_2_R in the neuroprotection and anti-neuroinflammation of PF, a specific CB_2_R antagonist AM630 was administered intraperitoneally 15 min prior to the PF administration each time.

### Morris water maze

The Morris water maze was provided by Beijing Zhongshidichuang Science and Technology Development Co., Ltd (Beijing, China), and different combinations of experimental apparatus were used to test positioning and navigation as well as spatial exploration abilities. Ten rats were randomly selected from each group for the water maze test. The water maze consisted of a circular pool of a diameter of 130 cm and a height of 50 cm, with a water depth of 30 cm and a water temperature of 26 ± 1 °C. At the walls of the maze, there were four labeled entry spots, dividing the maze into four quadrants. At the 4th quadrant, a platform with a diameter of 12 cm and a height of 29 cm was placed 1 cm below the water surface. The positioning and navigation experiment mainly tested the learning ability of the rats. On day 24 after drug administration, the rats in each group were placed into the pool at the four different entry points. The time the rats took to find and climb the platform (‘‘escape latency time’’) within 90 s was recorded, and the rats were continuously observed for 5 days. If the rats were unable to find the platform within 90 s, they were placed on the platform for 15 s and their escape latency period was recorded as 90 s. In addition, spatial exploration experiments were used to test the ability of the rats to remember the location of the platform. On day 5 when the positioning and navigation experiment ended, the platform was removed and the rats were placed into the 4th quadrant, into the water, facing the wall. The number of times that the rats crossed the original location of the platform within 120 s and the percentage of swimming routes within the 4th quadrant (relative to the total route) were recorded.

### Histopathological analysis

On the day after the water maze experiment ended, six rats were selected from each group and 45 mg/kg pentobarbital sodium was administered for anesthesia by intraperitoneal injection. Cardiac perfusion of 150 ml physiological saline was administered and 100 ml of 4% paraformaldehyde was used for fixation before the brains were extracted. A 3-mm-thick tissue block behind the optic chiasm was extracted and routine paraffin embedding was carried out. Continuous coronal sections (4 μm thick) were cut using a microtome (Leica Microsystems, Wetzlar, Germany) and stained with conventional hematoxylin and eosin (HE) staining. An Olympus BX51 light microscope was used to observe morphological and structural changes in the hippocampal CA1 region. The extent of the neuronal damage was quantified by counting the number of intact pyramidal neurons in the hippocampal CA1 area, which was expressed as a neuronal cell density.

### Electron microscopy observations

After rats in each group were anesthetized by intraperitoneal injection of 45 mg/kg pentobarbital sodium, 100 ml physiological saline was rapidly perfused into the left ventricular aorta. Then ice-cold fixing solution (4% paraformaldehyde in phosphate buffered saline; PBS) was perfused for 30 min. A sharp blade was used to rapidly resect the left hippocampal CA1 region and slice it into 1 × 1 × 1 mm^3^ hippocampal blocks. The blocks were immersed in 2.5% glutaraldehyde solution for 24 h. After that, 0.1 M PBS was used to wash the tissue blocks, and then 1% osmic acid was used for 1 h of fixation. An alcohol gradient was used for dehydration and the block was embedded in epoxy resin 618. After positioning and embedding of semithin sections, an ultramicrotome was used to process the slices into 60-nm ultrathin sections. The sections were collected in a polyvinyl formal-coated copper grid. Double staining with uranyl acetate and lead citrate was carried out. The ultrastructure of the hippocampal CA1 region of rats was observed under an H-7500 transmission electron microscopy (Hitachi, Tokyo, Japan) at 15,000× magnification.

### Enzyme-linked immunoassay (ELISA)

Six rats were selected from each group and rapidly decapitated for brain extraction. The hippocampus was rapidly isolated in an ice bath and blood was washed off the tissue surface using a 4 °C pre-cooled physiological saline. After blotting dry, the hippocampus was weighed and 4 °C pre-cooled physiological saline was added. An electric homogenizer was used to generate hippocampal homogenates with a 10% mass fraction. The homogenates were centrifuged at 3000 rpm for 10 min, and the supernatant was collected for assay. IL-1β, IL-6 and TNF-α concentrations in rat hippocampal tissues were measured using ELISA test kits (Shanghai JiJin Chemistry Technology Co. Ltd., Shanghai, China) following the manufacturer’s instructions. The detection limits for IL-1β, IL-6, and TNF-α were 0.1, 1 and 1 pg/ml, respectively. The concentration of each sample was calculated according to the corresponding standard curve and normalized to the protein concentration of the sample. The final unit used was pg/mg protein. Protein concentrations of the tissues were quantitated according to the bicinchoninic acid (BCA) assay kit (Beyotime). This method was used for subsequent protein concentration determination.

### Quantitation of nitrite concentrations

The NO concentration of rat hippocampal homogenates was indirectly measured by using the Griess reagent to measure the NO metabolite, nitrite (NO_2_^−^). The specific steps were as follows: 50 μl hippocampal homogenate and 50 µl Griess reagent (1% sulfanilamide, 0.1% *N*-[1-naphthyl-ethylenediamine dihydrochloride] and 0.1 M HCl) were allowed to react. Subsequently, a microplate reader (Thermo Electron Co., Waltham, MA, USA) was used to measure the optical density (OD) at 540 nm wavelength. The NO_2_^−^ concentration of the sample was calculated by plotting a standard NaNO_2_ curve (1–100 μM), and the unit used was nmol/g tissue.

### Western blotting analysis

Hippocampal specimens (30–50 mg) were removed from the − 80 °C freezer and 150–250 μl protein lysis buffer (Beyotime) and protease inhibitors (Roche Applied Science, Indianapolis, IN, USA) were added. The specimens were homogenized on ice and lysed for 30 min. In addition, nuclear lysates from tissues were obtained using the nuclear extraction kit (Pierce Biotechnology, Rockford, IL, USA). Equivalent amounts of protein samples (30 μg) were loaded on a 10–15% (w/v) SDS-PAGE for electrophoretic separation. The proteins in the gel were then transferred onto a polyvinylidene difluoride (PVDF; Millipore, Bedford, MA, USA) membrane and the membrane was blocked with 5% skim milk in Tris-buffered saline (TBS) containing 0.1% Tween 20 (TBS-T) at room temperature for 1 h. Subsequently, corresponding primary antibodies were added for incubation overnight at 4 °C. After washing thrice with TBS-T, the membranes were incubated at room temperature with anti-rabbit or anti-mouse secondary antibodies (Bioworld Technology) that were conjugated to horseradish peroxidase at a dilution of 1:10,000. The membranes were washed four times with TBS-T and visualized using enhanced chemiluminescence (ECL; Millipore). Images were obtained using the Amersham Imager 600 (GE Healthcare Biosciences, Little Chalfont, UK). The Quantity One software (Bio-Rad, Hercules, CA, USA) was used to analyze the integrated absorbance value of every band, with β-actin and lamin B1 being used as reference proteins for loading control in total cell lysates and nuclear extracts, respectively. The ratio of the integrated absorbance value of the target band in the sham-operated group and the reference protein band was set as 1. The ratio of the integrated absorbance value of target band in the different treatment groups and the reference protein band were compared to obtain the relative expression level of target proteins in each group.

### RNA reverse transcription and quantitative polymerase chain reaction (RT-qPCR)

Six rats were selected from each group and rapidly decapitated, and their hippocampi were rapidly isolated in an ice bath. Total RNA from the rat hippocampus was extracted using the RNApure high-purity total RNA rapid extraction kit (spin-column, BioTeke, Beijing, China) following the manufacturer’s instructions. Of the total RNA, 2 μg was used as a template, and Oligo (dT) was used as a primer with M-MuLV reverse transcriptase (BioTeke) in a 20 μl reaction system for reverse transcription to obtain cDNA. The cDNA was added into the SYBR FAST qPCR Master Mix (Kapa Biosystems, Woburn, MA, USA), together with primers for real-time quantitative PCR. Table 1 shows the primer sequences for rat CB_2_R, IL-1β, IL-6, TNF-α, IL-10, arginase-1, Ym1, TGF-β1 and β-actin. Representative cDNAs for various groups were mixed and a 5-fold dilution method was used to plot a standard curve to examine the qPCR amplification efficiency. The threshold cycle (Ct) is the number of the cycle in which the fluorescence signal becomes detectable above background. The Ct value is inversely proportional to the original template number. The comparative Ct method (2^−ΔΔCt^) was used for comparison of relative mRNA levels in different groups. Here, the ΔCt value represents the difference in Ct between the target genes in different groups and that of the internal reference gene β-actin. Subsequently, the ΔCt values in each treatment group were subtracted from those in the sham-operated group to obtain ΔΔCt.

**Table 1:** Primer sequences used for quantitative real-time PCR amplification

Gene	Forward primer (5′–3′)	Reverse primer (5′–3′)	Product length (bp)
CB_2_R	ACCTATGTCTGTGCTACCCACC	CCAGCCCAGGAGGTAGTCG	188
iNOS	AGTGGCAACATCAGGTCGG	CGATGCACAACTGGGTGAAC	166
IL-1β	ATGACCTGTTCTTTGAGGCTGAC	CGAGATGCTGCTGTGAGATTTG	114
TNF-α	GCCACCACGCTCTTCTGTC	GCTACGGGCTTGTCACTCG	149
IL-6	GTTGCCTTCTTGGGACTGATG	GCCATTGCACAACTCTTTTCTC	183
IL-10	GTTTTACCTGGTAGAAGTGATGCC	CCACTGCCTTGCTTTTATTCTC	155
Arginase-1	GGGAAAAGCCAATGAACAGC	CCAAATGACGCATAGGTCAGG	148
Ym1	TTGGAGGCTGGAAGTTTGG	AGGAGGGCTTCCACGAGAC	161
TGF-β1	GGCGGTGCTCGCTTTGTA	ATTGCGTTGTTGCGGTCC	135
β-Actin	CCCATCTATGAGGGTTACGC	TTTAATGTCACGCACGATTTC	150

### Immunofluorescence

After rats were decapitated, their brains were extracted and fixed in 4% paraformaldehyde at 4 °C for 24 h. The brains were then immersed in 30% sucrose solution overnight. Three-mm-thick tissue blocks in front of and behind the optic chiasm were extracted and cut into 14 μm thick cryosections. Subsequently, the sections were rinsed with PBS four times for 5 min each time. Then 10% goat serum was added for blocking at 37 °C for 1 h. In order to carry out staining with double fluorescence markers, the sections were co-incubated with mouse anti-CD68 monoclonal antibody and goat anti-CD206 polyclonal antibody (both 1:100 dilution) at 4 °C overnight. Subsequently, sections were further incubated with FITC-labeled goat anti-mouse and TRITC-labeled rabbit anti-goat IgG (both at a 1:100 dilution; Beijing Zhongshan Golden Bridge Biotechnology Co., Ltd., Beijing, China) at 37 °C for 1.5 h. After sufficient washing, in order to visualize cell nucleus, DAPI (Sigma Chemical Company, St. Louis, MO, USA) was added for nuclear staining for 10 min. Finally, stained sections were scanned and photographed with an A_1_R confocal laser scanning microscope (Nikon Instruments, Melville, Japan).

### Statistical analysis

Experimental data were collected and presented as mean ± standard deviation (SD). Two-way analysis of variance (ANOVA) was applied to compare the length of the escape latency period of rats. One-way ANOVA was used for intergroup comparisons of continuous variables. If the statistical analysis showed that differences between multiple groups were significant, Fisher’s least significant difference (LSD) post hoc analysis was employed for comparison of differences between two groups. All statistical analyses were carried out with SPSS version 19.0 software (IBM, Chicago, IL, USA), and a *P *< 0.05 was considered to be statistically significant.

## Results

### Influence of PF on behavior of the VD rat model

**Fig. 1:**
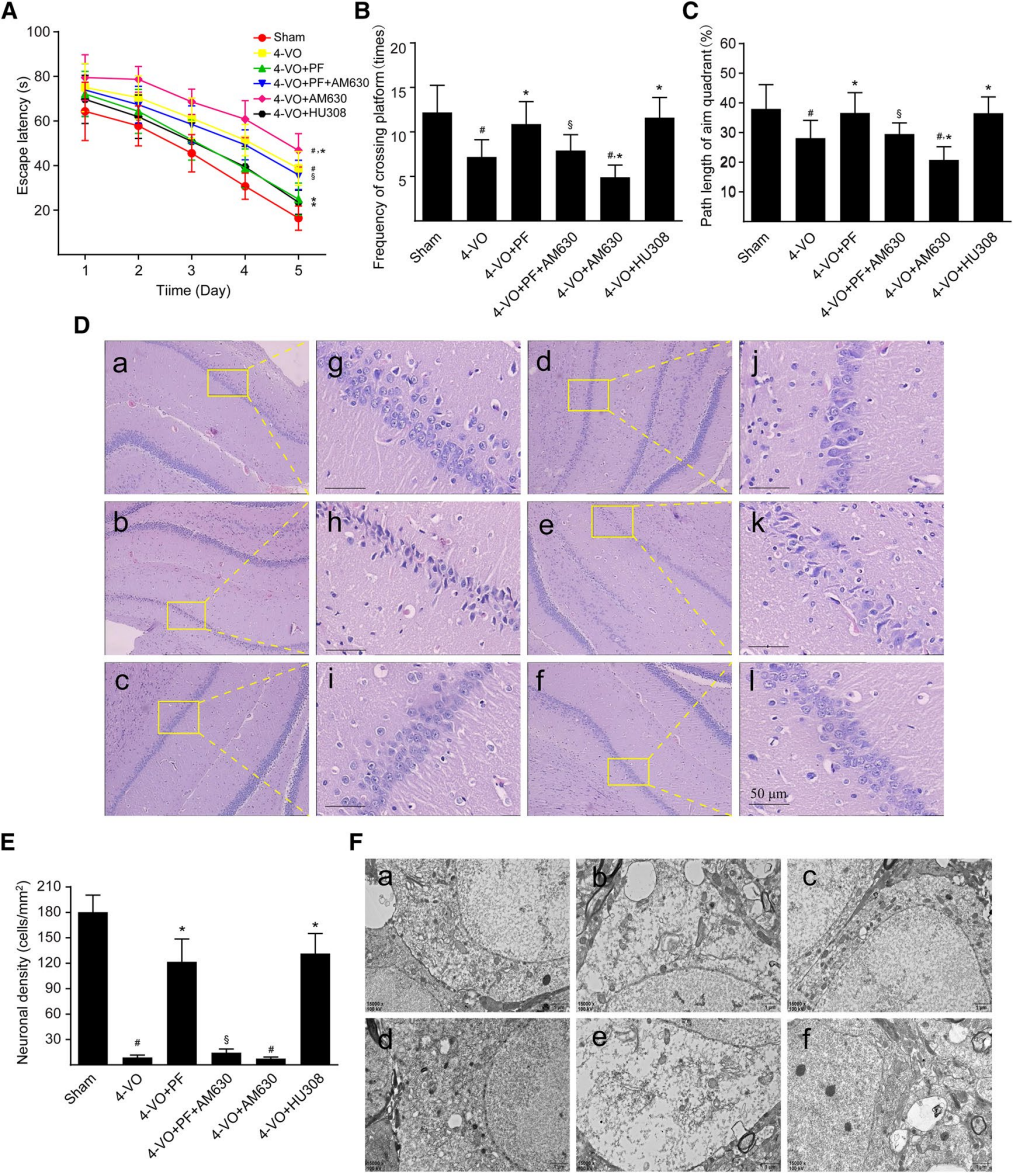
Effects of paeoniflorin on learning and memory abilities, histopathology and ultrastructure of the hippocampal CA1 area of rats after cerebral ischemia. One week after four-vessel occlusion (4-VO) surgery, rats were intraperitoneally administered saline (4-VO), paeoniflorin (4-VO+PF; 40 mg/kg/day), paeoniflorin+AM630 (4-VO+PF+AM630; 40 + 3 mg/kg/day), AM630 (4-VO+AM630; 3 mg/kg/day) or HU308 (4-VO+HU308; 3 mg/kg/day) for consecutive 28 days. Sham-operated group (Sham) was performed using the same surgical procedures, except that vertebral artery and bilateral common carotid arteries were not occluded. **A** Mean escape latency of rats in the water maze task was recorded during the last 5 days (23–28 days) of treatment. The spatial probe trial was performed on the last day (day 28) of water maze training, and the parameters measured included (**B**) the number of times rats crossed the platform and (**C**) relative path length in the target quadrant/path length in the whole pool within 120 s. **D** Rats were sacrificed after 28 days consecutive drug treatment. Representative photomicrographs of hematoxylin–eosin-stained hippocampal regions of rats are shown in different groups: (**a**, **g**) sham-operated group, (**b**, **h**) 4-VO-operated group, (**c**, **i**) 4-VO+PF group, (**d**, **j**) 4-VO+PF+AM630 group, (**e**, **k**) 4-VO+AM630 group or (**f**, **l**) 4-VO+HU308 group. Boxed regions in **a**–**f** are shown in **j**–**l**, respectively. Scale bar: 50 µm. **E** Neuronal cell density in CA1 region was measured by hematoxylin–eosin staining and cell counting. **F** Representative photomicrographs with a transmission electron microscope showing ultrastructural changes in hippocampus CA1 regions of rats in each group. Scale bar: 1 µm. Each bar represents mean ± SD of three independent experiments. *n* = 6 or 10 rats per group. ^#^*P *< 0.05 versus sham group, *P < 0.05 versus 4-VO model group, ^§^*P *< 0.05 versus 4-VO+PF group

Learning and memory deficits constitute some of the most injurious symptoms of VD. In behavioral research, the Morris water maze task is widely used for testing spatial learning and memory abilities in a VD rat model [26]. In the present study, rats in each group were subjected to the spatial navigation task at 24‒28 days after 4-VO (Fig. 1A) and showed decreased escape latencies over time, indicating that spatial learning and memory abilities improved with the prolong of training time. The escape latency of rats in the 4-VO model group was significantly longer than that in the sham operation group at each point during the training, indicating that the spatial learning ability of rats in the model group was severely impaired. However, compared with the model group, after the administration of PF or CB_2_R selective agonist HU308, the escape latency of rats in the model group was significantly shortened, and the reduction was most prominent from the 3rd to the 5th day of the training, demonstrating that PF and HU308 can significantly improve learning and memory abilities in rats following 4-VO. Compared with the PF group, the CB_2_R selective antagonist AM630 strikingly attenuated PF-induced decrease in escape latencies. Meanwhile, 4-VO rats treated with AM630 alone exhibited escape latencies even more prolonged than those seen in untreated 4-VO rats. Altogether, these results indicate that PF improves spatial learning and memory abilities in the 4-VO rat model of VD via the activation of CB_2_R.

When subjected to the spatial recognition test on the 28th day after surgery, 4-VO rats crossed the platform significantly fewer times and spent a significantly smaller percentage of their total path length swimming in the fourth target quadrant than did rats in the sham operation group (Fig. 1B, C), demonstrating that 4-VO rats had significantly impaired spatial memory. However, following treatment with PF or HU308, the number of times 4-VO rats crossed the platform and the percentage of their total path length spent swimming in the platform quadrant increased significantly, and this effect of PF was blocked when combined with AM630 treatment. When 4-VO rats received only AM630, they showed an even further reduction in the number of times of crossing the platform and spent an even smaller percentage of their total swimming path in the fourth target quadrant than did the untreated 4-VO rats. Taken together, these findings indicate that PF exerts a protective effect on spatial memory abilities in this rat model of VD and that this effect occurs via the activation of CB_2_R.

### Effects of PF on histopathology and ultrastructure of the hippocampal CA1 area after ischemia

The hippocampal CA1 region is closely associated with high-level cognitive functions, including learning and memory and is an area of the brain that is sensitive to ischemia [27]. Histopathological study with HE staining showed a large number of neurons with high density in the hippocampal CA1 region in the brain subjected to sham-operation. These neurons had abundant cytoplasm and large round nuclei with regular shapes (Fig. 1D [a, g]). In contrast, HE staining in 4-VO rats showed a significantly reduced number of pyramidal neurons in the hippocampal CA1 region (Fig. 1E), and most neurons had smaller cell bodies, a disordered arrangement, strongly stained eosinophilic cytoplasms, and irregular triangle-shaped and polygon-shaped nuclei (Fig. 1D [b, h]). Administration of PF (Fig. 1D [c, i] or HU308 (Fig. 1D [f, l]) preserved the normal structure of neurons in the hippocampal CA1 region and prevented the loss of nerve fibers. This protective effect of PF was blocked when 4-VO rats received AM630 in addition to PF (Fig. 1D [d, j]). Further, treatment with AM630 alone further decreased the number of neurons and nerve fibers compared with that in the untreated 4-VO group, and most neurons in these AM630-treated rats were smaller and exhibited strongly stained cytoplasm and irregular and darkly stained nuclei (Fig. 1D [e, k]). The histopathological changes in entorhinal cortex was well in agreement with those in the hippocampal CA1 region of rats (Additional file 3: Figure S2).

Transmission electron microscope imaging showed, in the sham group, the ultrastructure of hippocampal CA1 neurons was intact and clear; the nuclei were round and had a uniform distribution of chromatin and obvious nucleoli; the cytoplasm contained abundant organelles with clear structures; and the mitochondria could be clearly identified (Fig. 1F [a]). In contrast, CA1 in 4-VO rats had distended neuronal cell bodies, enlarged surrounding spaces, a reduced number of organelles surrounding the nucleus and otherwise generally disintegrated organelles. Specifically, the mitochondria appeared as swollen structures with both large vacuoles, and their cristae were disrupted and even disappeared (Fig. 1F [b]). Conversely, in 4-VO rats that received treatment with PF or HU308, the hippocampal CA1 neurons had a relatively intact and clear structure in which the chromatin was distributed evenly, the organelles in the cytoplasm were abundant, the mitochondrial membrane was largely intact, the mitochondria cristae were clear, and the endoplasmic reticulum was neatly arranged (Fig. 1F [c, f]). In these neurons, a small amount of chromatin condensation was occasionally observed, and only a few mitochondria were slightly swollen. However, when 4-VO rats were treated with both PF and AM630, the beneficial effects of PF were lost; the hippocampal CA1 neuronal ultrastructures lost integrity; and the area became disordered again, similar to that seen in untreated 4-VO rats (Fig. 1F [d]). Further, in 4-VO rats that received AM630 alone, the degrees of neuronal swelling, morphological damage, and structural disorder were exacerbated compared with those in the untreated 4-VO group (Fig. 1F [e]). Altogether, these results indicate that PF exerts a neuroprotective effect by alleviating neuropathological changes in the hippocampal CA1 region in a rat model of VD through the activation of CB_2_R.

### Effect of PF on CB_2_R expression and M1/M2 polarization markers in microglia/macrophages in the ischemic hippocampus

**Fig. 2:**
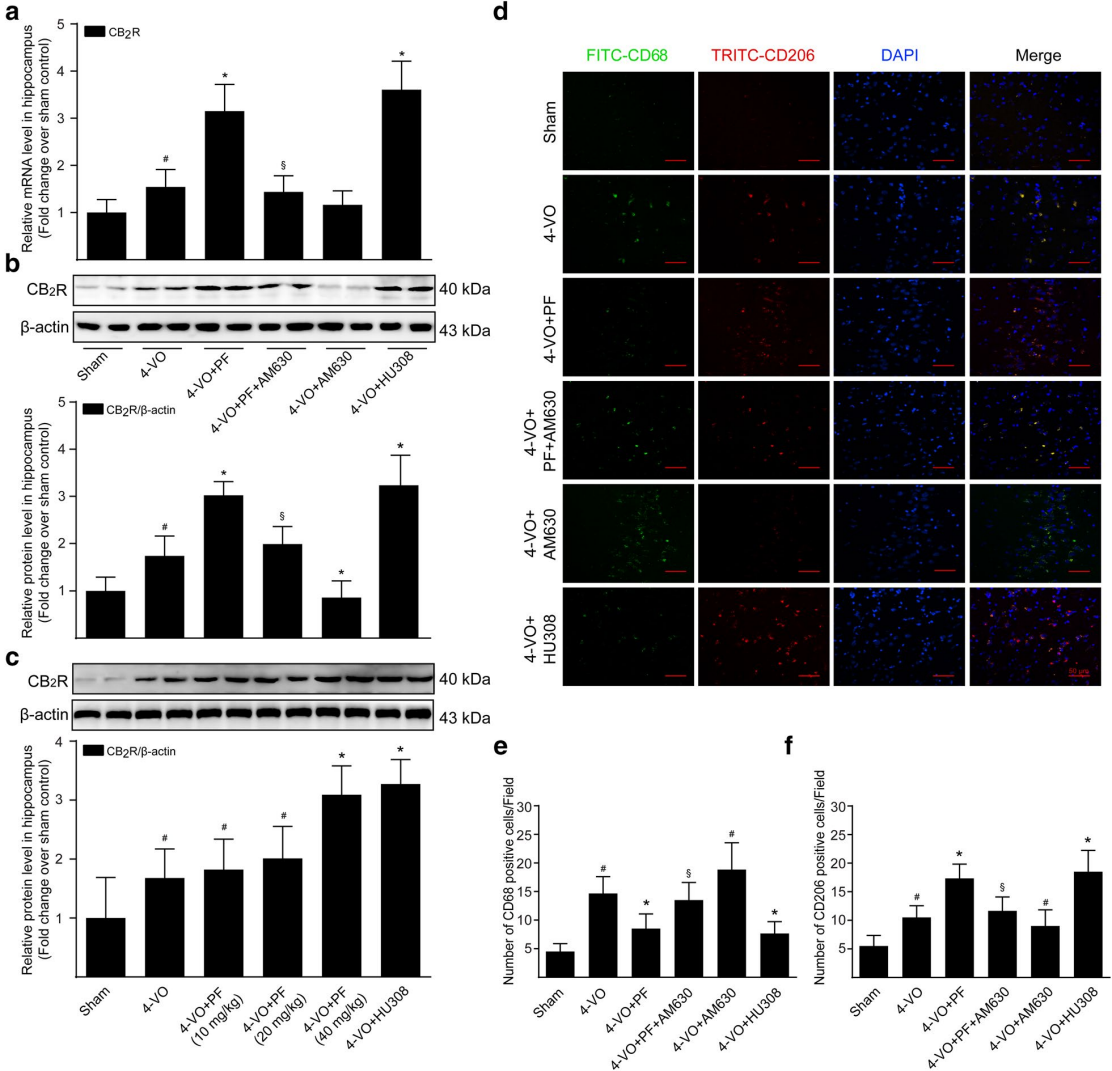
Effects of paeoniflorin on the expression of CB_2_R and M1/M2 polarization markers in microglia/macrophage in hippocampi of rats after cerebral ischemia. After 28 days consecutive drug treatment, **a** real-time quantitative PCR analysis of the mRNA level of CB_2_R in the hippocampus tissues of rats from different groups: sham (sham operated), 4-VO (four-vessel occlusion operated), 4-VO+PF (four-vessel occlusion operated plus paeoniflorin 40 mg/kg/day), 4-VO+PF+AM630 (four-vessel occlusion operated plus paeoniflorin 40 mg/kg/day and AM630 3 mg/kg/day), 4-VO+AM630 (four-vessel occlusion operated plus AM630 3 mg/kg/day) or 4-VO+HU308 (four-vessel occlusion operated plus HU308 3 mg/kg/day). **b** Proteins were extracted from hippocampi of rats and subjected to Western blotting analysis using primary antibody specific for CB_2_R. Blots were stripped and re-probed with antibody against β-actin to correct for differences in protein loading. Fold-change in the relative level of CB_2_R protein is shown after normalizing with β-actin. **c** CB_2_R protein level in hippocampi of rats was analyzed by Western blotting from different groups: sham (sham operated), 4-VO (four-vessel occlusion operated), 4-VO+PF (four-vessel occlusion operated plus paeoniflorin 10, 20, 40 mg/kg/day) or 4-VO+HU308 (four-vessel occlusion operated plus HU308 3 mg/kg/day). **d** Hippocampus tissue sections were co-stained for CD68 (M1 marker; green) or CD206 (M2 marker; red) and DAPI (blue). The images were observed and captured by a confocal laser scanning microscope. Scale bar: 50 μm. **e**, **f** Quantitative analysis of the number of CD68-positive and CD206-positive cells per visual field in the hippocampal CA1 region of rats. Each bar represents mean ± SD of three independent experiments. *n* = 6 rats per group. ^#^*P *< 0.05 versus sham group, *P < 0.05 versus 4-VO model group, ^§^*P *< 0.05 versus 4-VO+PF group

In ischemic brain tissue, activated microglia/macrophages are the primary participants in the neuroinflammatory response [28]. After cerebral ischemia, persistent inflammation in the brain can further aggravate neuronal injury. However, at the late phase of cerebral ischemia, microglia/macrophages undergo a phenotypic transition, adopting either an M1 or an M2 phenotype, which exert different physiological functions [29]. To examine whether PF can induce microglia/macrophages to transition from the proinflammatory M1 phenotype into the anti-inflammatory M2 phenotype by promoting CB_2_R expression, thereby reducing the inflammatory damage to peripheral neurons in a rat model of VD, we first measured the mRNA and protein expression levels of CB_2_R in the ischemic hippocampus, based on both RT-qPCR (Fig. 2a) and Western blotting (Fig. 2b, c) studies. The results showed compared with the sham operation group, hippocampal CB_2_R mRNA and protein expression levels were slightly elevated in rats of the 4-VO group. These methods also revealed that 4-VO rats that received PF showed even further increase in the expression of CB_2_R mRNA and protein, similar to that seen with HU308 treatment. Compared with the PF treatment alone, 4-VO rats that also received AM630 showed significantly reduced CB_2_R mRNA and protein levels, indicating that the CB_2_R antagonist AM630 significantly inhibits PF-mediated up-regulation of CB_2_R expression. Finally, 4-VO rats treated with AM630 alone showed significantly reduced CB_2_R mRNA and protein expression compared with untreated 4-VO rats. In addition, we also found that the density of Iba1^+^ cells per field in the ischemic hippocampi was significantly reduced in PF or HU308 treatment group compared with that in the 4-VO model group (Additional file 4: Figure S3). These results suggest that PF treatment suppresses the activation of microglia/macrophages in vivo.

Next, immunofluorescence double labeling was performed with M1 marker CD68 and M2 marker CD206 to evaluate the phenotypic polarization of microglia/macrophages in the ischemic hippocampus [28]. The results of this staining were consistent with the trend found for the expression of the CB_2_R. Specifically, compared with the sham operation group, the numbers of CD68-positive and CD206-positive microglia/macrophages in the hippocampal CA1 region of the 4-VO group both increased significantly (Fig. 2d). These two types of marker proteins co-localized on microglia/macrophages, suggesting that at later stages of ischemia some microglia/macrophages in the hippocampal CA1 region were undergoing the protective M1–M2 polarity transition response. However, treatment with either PF or HU308 further induced the M2 transition of hippocampal CA1 microglia/macrophages, visible as a reduced expression of the M1 marker (CD68) and a further increased expression of the M2 marker (CD206). When PF treatment was combined with AM630 in 4-VO rats, the PF-induced M2 transition of microglia/macrophages was blocked, and in 4-VO rats treated with AM630 alone, most microglia/macrophages in the hippocampal CA1 area were of the proinflammatory M1 phenotype, while the anti-inflammatory M2 marker (CD206) was hardly detectable. Altogether, these results suggest that PF induces microglia/macrophage polarization to an anti-inflammatory M2 phenotype in the hippocampus by up-regulating CB_2_R expression in a rat model of VD.

### Effect of PF on expression products of microglia/macrophages in the hippocampus after ischemia

**Fig. 3:**
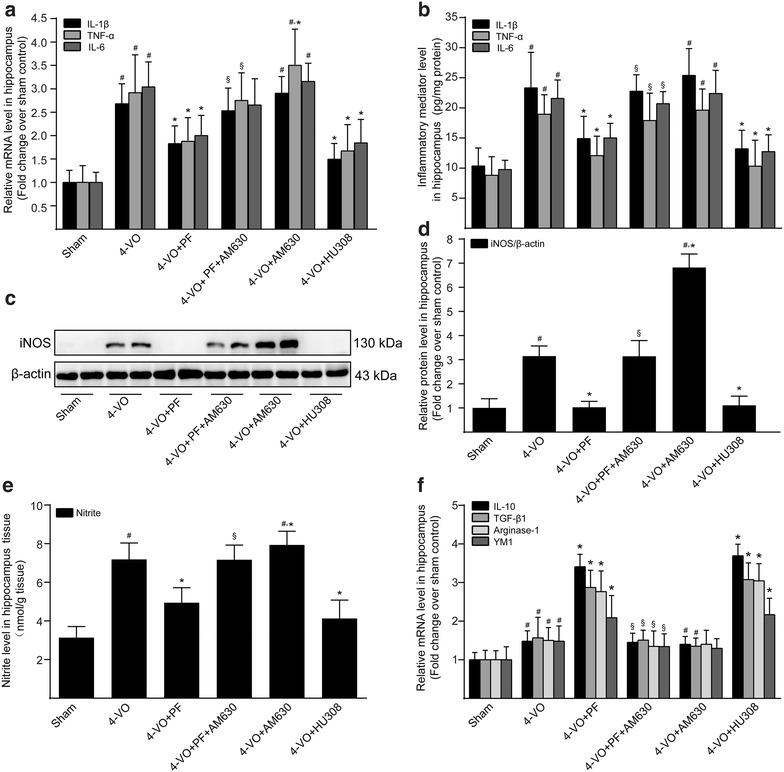
Effects of paeoniflorin on expression products of microglia/macrophage in hippocampi of rats after cerebral ischemia. One week after four-vessel occlusion (4-VO) surgery, rats were intraperitoneally administered saline (4-VO), paeoniflorin (4-VO+PF; 40 mg/kg/day), paeoniflorin+AM630 (4-VO+PF+AM630; 40 + 3 mg/kg/day), AM630 (4-VO+AM630; 3 mg/kg/day) or HU308 (4-VO+HU308; 3 mg/kg/day) for consecutive 28 days. **a** After treatment, real-time quantitative PCR analysis and **b** ELISA were used to detect the mRNA and protein levels of the proinflammatory cytokines IL-1β, TNF-α and IL-6, respectively. **c** Western blotting analysis was performed to further detect iNOS protein expression level. β-Actin served as an internal control. **d** The protein level of iNOS was expressed as arbitrary densitometric unit and normalized by the value of β-actin and finally expressed relative to the protein level in the sham-operated group (defined as 1-fold). **e** The content of nitrite in the hippocampus tissues of rats were determined by the Griess reaction. **f** Real-time quantitative PCR analysis was used to detect the mRNA levels of the anti-inflammatory cytokines IL-10 and TGF-β1; and the M2-associated markers arginase-1 and Ym1. Each bar represents mean ± SD of three independent experiments. *n* = 6 rats per group. ^#^*P *< 0.05 versus sham group, *P < 0.05 versus 4-VO model group, ^§^*P *< 0.05 versus 4-VO+PF group

After the ischemic injury, microglia/macrophages exhibit dynamic polarization over time, switching from the phenotype of alternatively activated M2 macrophages to an M1 profile [30]. M1-like microglia/macrophages mainly express proinflammatory proteins such as iNOS, IL-1β, TNF-α and IL-6, while M2-like microglia/macrophages mainly express anti-inflammatory proteins such as IL-10, arginase-1, TGF-β1 and Ym1. Compared with the sham operation group, the mRNA (Fig. 3a) and protein (Fig. 3b) levels of M1 expression products, including IL-1β, TNF-α and IL-6, were significantly increased in the hippocampal homogenate of the 4-VO group. Further, levels of the iNOS protein (Fig. 3c, d), a rate-limiting enzyme, and its inflammatory product, NO (Fig. 3e), were significantly increased in the hippocampal homogenate. Additionally, a significant induction of the M2-associated cytokines (IL-10 and TGF-β1) and M2-associated cell surface markers (arginase-1 and Ym1) was also found (Fig. 3f). Specifically, our data show that PF or HU308 markedly decreased the levels of IL-1β, TNF-α, IL-6, iNOS and NO, emphasizing the anti-inflammatory role of PF or HU308 in ischemic injury by inhibiting M1-microglia/macrophage polarization. On the other hand, the mRNA levels of IL-10, arginase-1, TGF-β1 and Ym1 were further increased by treatment with either PF or HU308. Treatment with PF and AM630 together blocked the effects of PF on microglia/macrophage M1/M2 polarization. Further, treatment with AM630 alone further increase proinflammatory mediator expression and NO production in the hippocampi of 4-VO rats compared with that seen in untreated 4-VO rats, but showed little effect on mRNA expression of anti-inflammatory products. Taken together, the above results suggest that PF attenuates the M1 phenotype and promotes M2 microglia/macrophage polarization by inducing CB_2_R expression.

### Effect of PF on the mTOR/NF-κB signaling pathway in the ischemic hippocampus

**Fig. 4:**
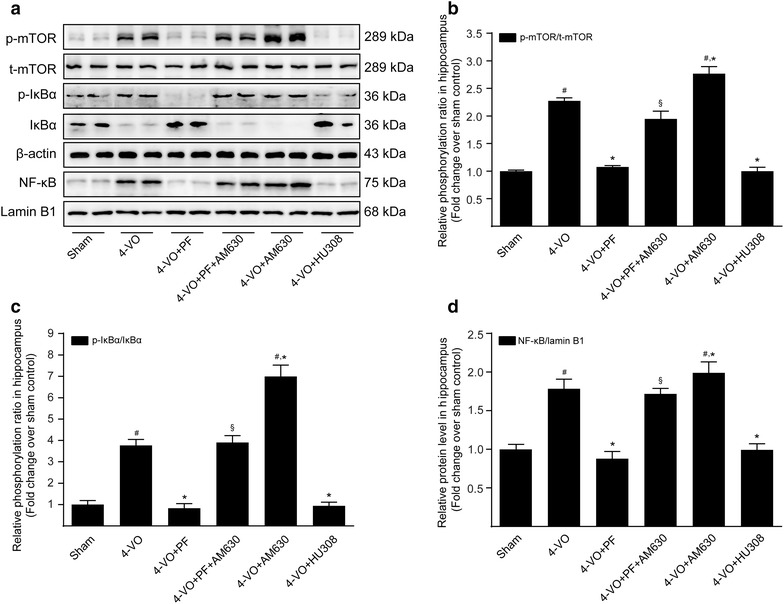
Effects of paeoniflorin on mTOR/NF-κB signaling pathway in hippocampi of rats after cerebral ischemia. One week after four-vessel occlusion (4-VO) surgery, rats were intraperitoneally administered saline (4-VO), paeoniflorin (4-VO+PF; 40 mg/kg/day), paeoniflorin+AM630 (4-VO+PF+AM630; 40 + 3 mg/kg/day), AM630 (4-VO+AM630; 3 mg/kg/day) or HU308 (4-VO+HU308; 3 mg/kg/day) for consecutive 28 days. **a** Representative Western blotting photographs showing protein levels of p-mTOR, mTOR, p-IκBα, IκBα and β-actin in cytoplasmic fractions and NF-κB p65 and lamin B1 in nuclear fractions of hippocampus tissues of rats. The β-actin and lamin B1 protein levels served as internal controls, respectively, for cytoplasmic extracts and nuclear extracts. Levels of phosphorylated **b** mTOR and **c** IκBα in cytoplasmic extracts, and of **d** NF-κB p65 in nuclear extracts, were converted to arbitrary densitometric units, normalized by the value of the corresponding loading control and expressed relative to the phosphorylation ratio or to the protein level in sham-operated group (defined as 1-fold). Each bar represents mean ± SD of three independent experiments. *n* = 6 rats per group. ^#^*P *< 0.05 versus sham group, *P < 0.05 versus 4-VO model group, ^§^*P *< 0.05 versus 4-VO+PF group

During the neuroinflammatory response, phosphorylated and activated mTOR induces phosphorylation and degradation of IκBα, leading to nuclear translocation of NF-κB and activation of the NF-κB signaling pathway [31]. Through this process, mTOR mediated the transcription of its regulated M1-associated genes and accelerates the process of neuroinflammation. Western blot analysis of hippocampal tissue showed that, compared with the sham operation group, 4-VO rats had significantly increased phosphorylation of mTOR (Fig. 4a, b) and IκBα (Fig. 4a, c) in cytoplasmic extracts and NF-κB p65 (Fig. 4a, d) in nuclear extracts, and they had significantly reduced expression of IκBα (Fig. 4a, c) in cytoplasmic extracts. Compared with untreated 4-VO rats, treatment with either PF or HU308 significantly reduced hippocampal levels of p-mTOR and p-IκBα protein in cytoplasmic extracts and of NF-κB p65 protein in nuclear extracts, and at the same time increased levels of IκBα in the cytoplasm. Treatment with both AM630 and PF together in 4-VO rats blocked effects seen with PF treatment alone, while treatment with AM630 alone in 4-VO rats further increased the expression levels of p-mTOR and p-IκBα protein in cytoplasmic extracts of the hippocampus, accelerated degradation of IκBα, and increased levels of NF-κB p65 protein in nuclear extracts. Altogether, these results indicate that PF blocks the mTOR-regulated NF-κB signaling pathway by activating expression of the CB_2_R in microglia/macrophages, and thereby inhibits the nuclear translocation of NF-κB and, ultimately, the transcription of M1-related inflammatory products.

### Effect of PF on the PI3K/Akt signaling pathway in the ischemic hippocampus

**Fig. 5:**
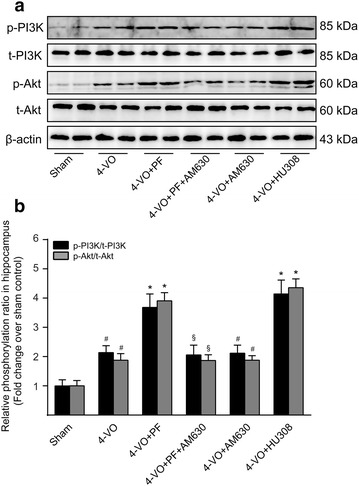
Effects of paeoniflorin on PI3K/Akt signaling pathway in hippocampi of rats after cerebral ischemia. One week after four-vessel occlusion (4-VO) surgery, rats were intraperitoneally administered saline (4-VO), paeoniflorin (4-VO+PF; 40 mg/kg/day), paeoniflorin+AM630 (4-VO+PF+AM630; 40 + 3 mg/kg/day), AM630 (4-VO+AM630; 3 mg/kg/day) or HU308 (4-VO+HU308; 3 mg/kg/day) for consecutive 28 days. **a** After treatment, whole tissue lysates of hippocampus were prepared and subjected to Western blotting analysis using antibodies specific for phosphorylated forms or all forms of PI3K and Akt. β-Actin was used as a loading control. **b** The protein levels of phosphorylated PI3K and Akt were converted to arbitrary densitometricunits, normalized by the value of the corresponding total protein level and expressed relative to the phosphorylation ratio in sham-operated rats (defined as 1-fold). Each bar represents mean ± SD of three independent experiments. *n* = 6 rats per group. ^#^*P *< 0.05 versus sham group, *P < 0.05 versus 4-VO model group, ^§^*P *< 0.05 versus 4-VO+PF group

Accumulative evidence suggests activation of the PI3K/Akt signaling pathway is involved in microglia/macrophage M2 polarization [32]. To evaluate whether PF alters M2-associated gene expression by increasing phosphorylation and activation of PI3K/Akt, hippocampus tissue lysates were prepared and subjected to Western blotting analysis using antibodies specific for PI3K and Akt, and their phosphorylated forms. The results showed that the phosphorylated levels of PI3K and Akt in the hippocampi of 4-VO rats were significantly higher than those in the sham-operated control (Fig. 5a, b). After treatment with either PF or HU308, the phosphorylation of PI3K and Akt in the hippocampus was further increased, whereas this effect of PF could be attenuated by combined treatment with AM630. Notably, 4-VO rats treated with AM630 alone did not show significantly different levels of phosphorylated PI3K and Akt in the hippocampus than that seen in the untreated 4-VO group. In all cases, total PI3K and Akt protein levels did not change greatly. These results indicate that that PF activates the PI3K/Akt signaling pathway and induces transition to the anti-inflammatory M2 phenotype by up-regulating CB_2_R expression in microglia/macrophages.

## Discussion

Microglia/macrophage-mediated neuroinflammation is a common component of neurodegenerative disorders, including Alzheimer’s disease, Parkinson’s disease, multiple sclerosis and VD. Chronic activation of microglia/macrophages often occurs during early stages of these CNS inflammatory diseases. Recent studies have found that sustained activation of microglia/macrophages is not simply the result of neuropathological changes, but rather is an active contributor to these changes, as activated microglia/macrophages (M1 phenotype) can synthesize and secrete large amounts of reactive oxygen and nitrogen species, proinflammatory cytokines (e.g., IL-1, IL-6, TNF-α and NO) and proteases, can damage neuronal cells, and can participate in the progression of chronic neurodegenerative diseases [33]. In addition, when the CNS is affected by certain pathological changes, such as ischemia and inflammation, an alternatively activated M2 program is initiated. Alternative activated microglia/macrophages (M2 type) can exert functions of phagocytosis, remove damaged cell debris, and secrete neurotrophic factors and anti-inflammatory mediators (e.g., IL-10, TGF-β and brain-derived neurotrophic factor), thus playing dual roles of anti-inflammation and neuroprotection [34]. Therefore, from a therapeutic perspective, simply inhibiting all microglia/macrophage activity is not the best therapeutic option in VD. Selectively blocking the proinflammatory effects of microglia/macrophages and simultaneously inducing their transition to the phenotype of neuroprotection and regeneration may have more profound therapeutic effects.

CB_2_R plays a key role in the regulation of inflammation and autoimmune diseases [35, 36]. Previous studies in primary rat cell cultures have reported that activation of CB_2_R can obviously inhibit the LPS-induced transition of microglia to the Ml phenotype and thereby prevent M1-mediated inflammatory responses [37]. Further, in an animal model of VD, the selective activation of CB_2_R improves memory deficit and infarct size in chronic brain hypoperfusion [38]. In support of these reports, our findings demonstrated that in the VD model rats which received either PF or a specific pharmacological CB_2_R agonist HU308, hippocampal CB_2_R mRNA and protein expression levels in 4-VO rats at the recovery stage of ischemia were further increased compared with that in the untreated 4-VO group. More importantly, PF or HU308 could also suppress the M1 proinflammatory microglia/macrophage phenotype while enhance the M2 repair phenotype, characterized by lower production of IL-1, IL-6, TNF-α and iNOS, higher expression of TGF-β1 and IL-10 and had slighter damage to hippocampal neurons and nerve fibers, thereby exhibiting anti-inflammatory and neuroprotective effects in the hippocampus of rats. The M1–M2 transition of microglia/macrophages regulated by PF could be blocked by a selective CB_2_R antagonist AM630. Taken together, the above results suggest that PF could antagonize toxic M1 responses and enhance protective M2 responses in microglia/macrophages following cerebral ischemia by activating CB_2_R and simultaneously by up-regulating CB_2_R expression, thereby protecting neighboring neurons indirectly.

The CB_2_R is a G protein-coupled receptor widely distributed in the brain. Multiple downstream signaling molecules mediated by the CB_2_R are involved in the modulation of microglia/macrophage polarity transition [39]. Among these, NF-κB is considered to be one of the most critical regulators in the activation of the microglia/macrophage M1 phenotype by controlling the transcription of M1-associated genes [40]. In the inactivated state, NF-κB is retained in the cytoplasm by binding to an inhibitory protein (IκB), but when microglia/macrophage cells are stimulated by inflammation, IκB is phosphorylated and polyubiquitinated, followed by degradation by the 26S proteasome, resulting in the dissociation of IκB from NF-κB. Subsequently, the released NF-κB translocates into the nucleus, where it binds to its cognate DNA sequence and regulates the transcription of various inflammatory mediators involved in M1 polarization, such as IL-1, IL-6, TNF-α and iNOS. At the same time, in response to the inflammatory response, the mTOR signaling pathway is also activated by phosphorylation, which can accelerate the development of inflammation through activating the NF-κB signaling pathway, resulting in ischemic necrosis of neurons. Consistent with a previous study by Li et al. who showed that administration of *trans*-caryophyllene, a CB_2_R selective agonist, efficiently attenuates hypoxia-induced neuroinflammatory response through the NF-κB pathway in microglia [41]. Our study demonstrated that in 4-VO rats, treatment with PF or HU308 prominently inhibited the phosphorylation of mTOR and IκBα, reduced IκBα degradation, and further blocked the nuclear translocation of the NF-κB p65 subunit, thereby significantly inhibiting the expression of markers for M1 microglia/macrophages. However, this inhibitory effect of PF on mTOR/NF-κB signaling could be abolished by the simultaneous administration of AM630. Our findings suggest that PF inhibits mTOR/NF-κB signaling pathway in the hippocampus of 4-VO rats by up-regulating the CB_2_R expression, thereby reducing the impact of the M1 microglia/macrophage phenotype and attenuating neuroinflammation. It is hard to conclude that the effect is derived from microglia rather than other cells, such as astrocytes and endothelial cells, even blood cells. Meanwhile, we conducted in vitro experiments to elucidate the effect of PF is derived from microglial cells rather than other cells. Our data from expeiments in vitro demonstrate that activation of CB_2_R by PF facilitates the microglia phenotype switch from proinflammatory (“M1-like”) to anti-inflammatory and immunomodulatory (“M2-like”) via suppressing the mTOR/NF-κB signaling pathway (Additional file 5: Figure S4).

The PI3K/Akt signaling pathway is widely present in various cell types. This pathway is involved in the regulation of cell growth, proliferation and differentiation through a series of responses and is a classic anti-apoptotic and pro-survival signal transduction pathway. In recent years, studies have shown that the CB_2_R agonist exhibits a neuroprotective effect in the CNS by activating the PI3K/Akt signaling pathway [42], which reduces cognitive impairment, increases cerebral blood flow, and inhibits neuronal apoptosis in an animal model of VD [43]. Akt is a common upstream regulator of both mTOR and NF-κB pathways. Akt activation directly phosphorylates mTOR and activates both IκB kinase and its downstream target, NF-κB. However, recent studies have found that everolimus, a small molecule mTOR inhibitor, can increase the phosphorylation of Akt and directed local microglia towards an anti-inflammatory state by shifting to an increased M2 profiles. It has been postulated that this effect may be related to the ability of everolimus to block the negative feedback effect of mTOR on Akt [10]. Moreover, PF was found to activate the PI3K/Akt signaling pathway, up-regulate Akt phosphorylation, and exert neuroprotective effects [44]. In the present study, the hippocampal phosphorylation of PI3K and Akt in 4-VO rats increased significantly with PF treatment. Meanwhile, mRNA levels of M2 microglia expression products (IL-10, arginase-1, Ym1 and TGF-β1) were also significantly increased. After treatment with both PF and the CB_2_R selective antagonist, AM630, the activation effect of PF on the PI3K/Akt pathway was significantly inhibited. Therefore, we hypothesize that PF increases the phosphorylation levels of PI3K/Akt by up-regulating CB_2_R expression, thereby inducing the transition of microglia/macrophages to the M2 phenotype, stimulating the secretion of neurotrophic factors and promoting brain repair and regeneration.

This present study has some limitations. First, neuroinflammation is reflected in VD model as astrogliosis and microglia activation [45]. Although CB_2_R activation by PF on the proinflammatory microglial cells might play a vital role to ameliorate neuronal death after cerebral ischemia, we did not exclude the possibility that PF could inhibit astrogliosis after ischemic brain damage by activating CB_2_R. Second, in the brain CB_2_R is expressed in activated astrocytes, microglial cells, neurons and endothelial cells [46], we only focused on the anti-neuroinflammatory mechanisms of CB_2_R activation by PF in activated microglia/macrophages. However, other mechanisms of CB_2_R stimulation in different brain cells might exist and play a role in providing beneficial effects after PF administration, which we did not explore in this study.

## Conclusion

The main findings of this study are as follows: PF exerts anti-neuroinflammation functions in a rat model of VD by specifically inducing CB_2_R function in microglia/macrophages of the hippocampus and consequently reducing the activity of the mTOR/NF-κB signaling pathway, thereby inhibiting the M1 microglia/macrophage release of proinflammatory factors. Further, the present findings suggest that PF activates the PI3K/Akt signaling pathway in a CB_2_R-dependent manner to promote the transition of microglia/macrophages from the M1 to M2 phenotype and stimulate M2 microglia/macrophages to secrete anti-inflammatory molecules and neuroprotective factors, thus reducing neuronal damage and further exerting neuroprotective functions. Our findings suggest that PF could be a potential therapeutic agent for the treatment of neuroinflammation-related CNS diseases, particularly VD.

## Additional files

**Additional file 1.** The Minimum Standards of Reporting Checklist.

**Additional file 2: Figure S1.** Effects of paeoniflorin on histopathology and protein levels of proinflammatory cytokines in the hippocampal CA1 area of rats after cerebral ischemia. One week after four-vessel occlusion (4-VO) surgery, rats were intraperitoneally administered saline (4-VO), paeoniflorin (4-VO+PF; 10, 20, 40 mg/kg/d) or HU308 (4-VO+HU308; 3 mg/kg/d) for consecutive 28 days. (A) Rats were sacrificed after 28 days consecutive drug treatment. Representative photomicrographs of hematoxylin-eosin-stained hippocampal regions of rats are shown in different groups: (a, g) sham-operated group, (b, h) 4-VO-operated group, (c, i) 4-VO+10 mg/kg/d PF group, (d, j) 4-VO+PF+20 mg/kg/d group, (e, k) 4-VO+40 mg/kg/d group or (f, l) 4-VO+3 mg/kg/d HU308 group. Boxed regions in a–f are shown in j–l, respectively. Scale bar: 50 µm. (B) Neuronal cell density in CA1 region was measured. (C) The protein levels of proinflammatory cytokines including IL-1β, TNF-α and IL-6 in the hippocampal homogenate were measured by enzyme linked immunosorbent assay.

**Additional file 3: Figure S2.** Effects of paeoniflorin on histopathology of the entorhinal cortex area of rats after cerebral ischemia. Representative photomicrographs of hematoxylin-eosin-stained entorhinal cortex region of either sham-operated rats (a, g) or rats that had been subjected to four-vessel occlusion followed by the treatment with saline (4-VO; b, h), paeoniflorin (4-VO+PF; 40 mg/kg/d; c, i), paeoniflorin+AM630 (4-VO+PF+AM630; 40 + 3 mg/kg/d; d, j), AM630 (4-VO+AM630; 3 mg/kg/d; e, k) or HU308 (4-VO+HU308; 3 mg/kg/d; f, l) for consecutive 28 days. Boxed regions in a–f are shown in j–l, respectively. Scale bar: 50 µm.

**Additional file 4: Figure S3.** Effects of paeoniflorin on the activation of microglial cells in hippocampi of rats after cerebral ischemia. One week after four-vessel occlusion (4-VO) surgery, rats were intraperitoneally administered saline (4-VO), paeoniflorin (4-VO+PF; 40 mg/kg/d), paeoniflorin+AM630 (4-VO+PF+AM630; 40 + 3 mg/kg/d), AM630 (4-VO+AM630; 3 mg/kg/d) or HU308 (4-VO+HU308; 3 mg/kg/d) for consecutive 28 days. After treatment, hippocampus tissue sections were co-stained for Iba1 (an activated microglia marker; green) or DAPI (blue). The images were observed and captured by a confocal laser scanning microscope. Scale bar: 50 μm.

**Additional file 5: Figure S4.** Effects of PF on M1/M2 polarization and mTOR/NF-κB signaling pathway in BV-2 microglia exposed to oxygen glucose deprivation (OGD). BV-2 cells were pre-incubated with DMSO (vehicle), PF (50 μM), AM630 (2 μM) or HU308 (10 μM) for 4 h followed by OGD for 6 or 24 h. (A) The cell lysates were immunoblotted with iNOS or β-actin antibody. β-Actin served as a loading control. These results are representative for three independent experiments. (B) The differences of the protein expression between the groups were analyzed with Image J. (C) The levels of nitrite in cell culture supernatants were determined by the Griess reaction. (D) Representative Western blotting photographs showing protein levels of p-mTOR, mTOR, p-IκBα, IκBα, NF-κB and β-actin in cytoplasmic fractions and NF-κB and lamin B1 in nuclear fractions of BV-2 microglia. The β-actin and lamin B1 protein levels were used as internal controls, respectively, for cytoplasmic extracts (CE) and nuclear extracts (NE). (E, F) The protein levels of phosphorylated and total mTOR and IκBα in cytoplasmic extracts and NF-κB in cytoplasmic and nuclear extracts, were converted to arbitrary densitometric units, normalized by the value of the corresponding loading controls and expressed relative to the phosphorylation ratio or to the protein levels in vechile control (defined as 1-fold). (G, H) The levels of IL-1β, IL-6, TNF-α, IL-10 and TGF-β1 in cell culture supernatants were determined by enzyme-linked immunosorbent assay. Each bar represents mean ± SD of three independent experiments. ^#^*P* < 0.05 versus vehicle control group, *P < 0.05 versus OGD group, ^§^*P* < 0.05 versus OGD+PF group.

## Abbreviations

4-VO: four-vessel occlusion
BCA: bicinchoninic acid
CB_1_R: cannabinoid receptor 1
CB_2_R: cannabinoid receptor 2
CNS: central nervous system
Ct: threshold cycle
HE: hematoxylin and eosin
iNOS: inducible nitric oxide synthase
LPS: lipopolysaccharide
mTOR: mammalian target of rapamycin
NO: nitric oxide
NF-κB: nuclear factor-κb
PBS: phosphate buffered saline
PI3K: phosphatidylinositol-3-kinase
PF: paeoniflorin
TNF-α: tumor necrosis factor-α
VD: vascular dementia
