# Supplementary material for: Paeoniflorin exerts neuroprotective effects by modulating the M1/M2 subset polarization of microglia/macrophages in the hippocampal CA1 region of vascular dementia rats via cannabinoid receptor 2
Source: Chin Med. 2018 Mar 20;13:14. doi: 10.1186/s13020-018-0173-1 (PMC5859430; doi:10.1186/s13020-018-0173-1)
Supplement: Supplementary file 1 — Additional file 1. The Minimum Standards of Reporting Checklist. [file 13020_2018_173_MOESM1_ESM.pdf]

Minimum Standards of Reporting Checklist

BioMed Central advocates full and transparent reporting. Please ensure thatyourpaper provides the information requested below where applicable. Onsubmittingyour paper you will be asked to confirm you have included this information, orgivereasons for any instances where it is not made available. You will also be askedtoupload this file and it should be cited in the Methodssection.

Experimental design andstatistics

The following information should be included in the Methods section and inserted in the table below:

**Question**	**Answer**
1. The exact sample size (n) for each experimental group/condition (as a number, not a range). Include details of a power analysis if done, or any other relevant considerations that determined the choice of sample size. For n < 6, individual data values should be shown rather than summary statistics alone.	The exact sample size (n) for each experimental group is as follows: Fig. 1A-C (n=10); Fig. 1D, E (n=6); Fig. 2 (n=6); Fig. 3 (n=6); Fig. 4 (n=6); Fig. 5 (n=6).<br>After verifying the distribution of the data by Kolmogorov-Smirnov test, the two-way analysis of variance (ANOVA) was used to compare the length of the escape latency period of rats. One-way ANOVA was used for intergroup comparisons of continuous variables, and the value was expressed as means ± SD.<br>This information is included in the manuscript.

2. A description of sample collection that enables the reader to understand whether the samples represent technical or biological replicates, and an explanation of inclusion/exclusion criteria if samples or organisms were excluded from the analysis.	Rats were subjected to the Morris water maze test, and rats that failed to find the platform within 90 s were excluded.<br>For Morris water maze, 120 rats of dementia were selected, based on the results from the Morris water maze with the following success criterion: using the mean value of the time taken by sham-operated rats to escape from the maze as a reference value, rats with a ratio of the difference between the escape latency period and the reference value greater than 20% were defined as dementia rats.<br>For histopathological analysis, a tissue block 3 mm behind the optic chiasm was extracted and routine paraffin embedding was carried out.
	For electron microscopy observations, a sharp blade was used to rapidly resect the left hippocampal CA1 region and slice it into $1\times1\times1\ mm^3$ hippocampal blocks.<br>For ELISA, six rats were selected from each group and rapidly decapitated for brain extraction. The hippocampus was rapidly isolated in an ice bath.<br>For RT-qPCR analysis, six rats were selected from each group and rapidly decapitated, and their hippocampi were rapidly isolated in an ice bath.<br>For Western blotting analysis, hippocampal specimens (30–50 mg) were removed from the -80 ℃ freezer and 150–250 μL protein lysis buffer and protease inhibitors were added.

3. How samples/ organisms were allocated to experimental groups and processed, and full details of the randomisation procedure used (if relevant).	One week after surgery, 120 successful dementia rat models were selected, based on the results from the Morris water maze with the following success criterion: using the mean value of the time taken by sham-operated rats to escape from the maze as a reference value, rats with a ratio of the difference between the escape latency period and the reference value greater than 20% were defined as dementia rats. In addition to the sham-operated group (n=24), successful models were randomly assigned to five groups (n=24): the model group, the PF (40 mg/kg) group, the PF (40 mg/kg) + AM630 (3 mg/kg) group, the AM630 (3 mg/kg) group, and the HU308 (3 mg/kg) group
4. For sample assessment by human investigators, a statement on whether the investigator was blinded to group assignment and outcome assessment, and how this blinding was achieved and evaluated (if relevant).	Not applicable.

5. How many times each experiment shown was replicated and an indication of the extent of variation from experiment to experiment.	This information has been included in the "Materials and Methods" section.
6. Information on the statistical methods and measures used. It should be clear whether the tests are one-sided or two-sided, whether there are adjustments for multiple comparisons, whether medians or means are being shown, whether error bars are standard deviations (SD), standard error of mean (SEM)or confidence intervals.	Two-way analysis of variance (ANOVA) was used to compare the escape latency period of rats in Fig. 1A. One-way ANOVA was used for intergroup comparisons of continuous variables. If the statistical analysis showed that differences between multiple groups were significant, Fisher's least significant difference (LSD) post hoc analysis was employed for comparison of differences between two groups. Data were expressed as mean ±standard deviation (SD).
7. A justification for the appropriateness of statistical tests used to assess significance. Do the data meet the assumptions of the tests? Is there an estimate of variation within each group of data, and is the variance similar between groups that are being statistically compared? In addition, information essential to interpreting the data presented should be made available in the figure and table legends. If the study involves health interventions for human participants, please refer to the relevant reporting guidelines from the EQUATOR Network, and the Biosharing Portal for reporting checklists for biological and biomedical research, where applicable.	Yes, there is an estimate of variation within each group of data. When the variance is significantly similar or different between groups, Kolmogorov-Smirnov test was used. A value of $P<0.05$ was considered statistically significant difference.<br><br>Information essential to interpreting the data was added in the figure legends.<br><br>This experiment is performed only with animal model but not with human participants.

BioMed Central
The Open Access Publisher

Research involving humans

If your research involved humans, please confirm you have adhered to therelevantreporting guideline from the EQUATOR Network, and included thecompletedchecklist as an additional file with yoursubmission:

	**Answer** (page and linenumberinserted/Not
- I have followed the relevant reporting for my study type,and included a populated checklist with my submission<br>- Not applicable for my study	Not applicable for my study.

Resources

A description of all resources used should be included in the Methods section,with enough information to allow them to be uniquely identified. The table below should be completed with confirmation that this was done (i.e. included in the Method ssection) or is not applicable. If this has not been completed, but is applicable, you should contact the journal editorial staff before proceeding.

	**Answer** (page and linenumberinserted/Not applicable formystudy)
•Antibodies: report source, catalogue code,characteristics, dilutions and how they were validated for the system understudy.	Page 9,18 and 19.
•Cell lines: report source, whether identity has been authenticated and whether tested for my coplasma contamination.We encourage researchers to check the NCBI database for contamination of celllines.	Not applicable for my study. We did not use cell line.
•Organisms: report source, species,strain,sex, age, husbandry, inbred and strain characteristics of transgenic and mutant animals.	Male Sprague-Dawley (SD) rats.
•Tools (software, databases andservices):report standard tool name, provider and version number, if available. For antibodies,model organisms (mice, zebrafish andflies)and tools, authors are strongly encouraged to cite Research Resource Identifiers (RRIDs).To do so, please go to the Resource Identification Portal to search for your research resource and insert the reference text into your Methods section.	All statistical analyses were carried out with SPSS version 19.0 software (IBM, Chicago, IL, USA). We did not use specialized tool but use commercial equipments.

BioMed Central
The Open Access Publisher

Availability of data and materials

The table below should be completed with confirmation that this was done(i.e.included in the Methods section) or is not applicable.

	**Answer** (page and linenumberinserted/Not applicable formystudy)
All data sets on which the conclusions of the paper rely must be either deposited inpublicly available repositories (where available and ethically appropriate) or presented in the main paper or additional supporting files,inmachine-readable for mat when ever possible. If authors are unable to fulfil this requirement, they should contact journal editorial staff,after checking our list of Recommended Repositories.	Not applicable for my study.
Links to deposited data sets, or data sets in additional files, should beexplicitlyreferenced in a section entitled "A vailability of Data and Materials". Guidance on where to deposit your data can be found on the Availability of Data and Materials policypage.	Not applicable for my study.
If computer code was used to generate results that are central to the paper'sconclusions,include a statement in the "Availability ofdataand materials" section to indicate howthecode can be accessed. Includeversioninformation and any restrictionsonavailability. For deposited data and published code, a full reference with an accession number, doi or other unique identifier should be included in the reference list.	Not applicable for my study.
If reproducible materials are generated asaresult of the research (for example new animal mutants), a statement on their availability should be included.	Not applicable for my study.
