# Supplementary figures and images for: Paeoniflorin exerts neuroprotective effects by modulating the M1/M2 subset polarization of microglia/macrophages in the hippocampal CA1 region of vascular dementia rats via cannabinoid receptor 2
Source: Chin Med. 2018 Mar 20;13:14. doi: 10.1186/s13020-018-0173-1 (PMC5859430; doi:10.1186/s13020-018-0173-1)

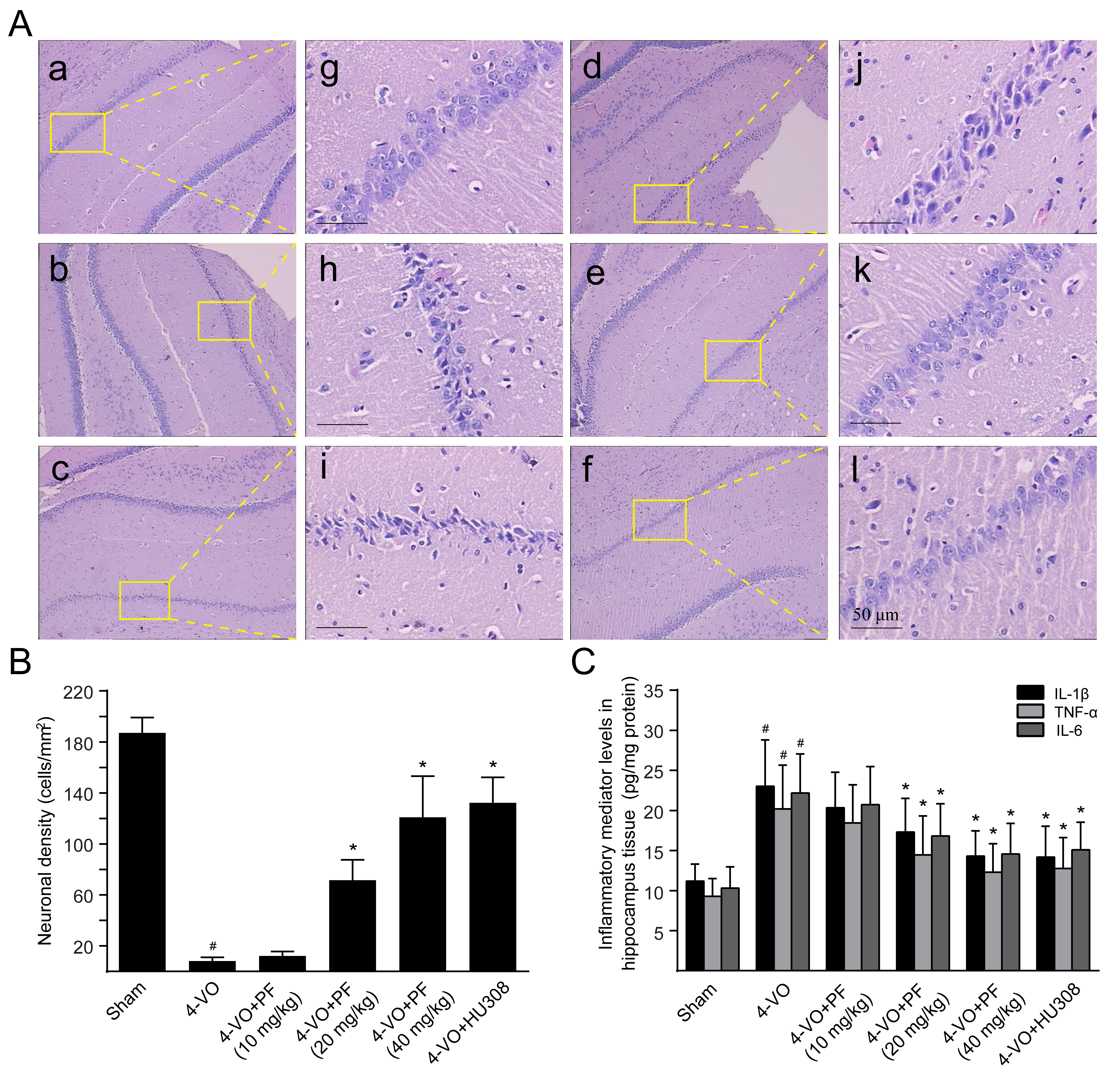

Supplement: Supplementary file 2 — Additional file 2: Figure S1. Effects of paeoniflorin on histopathology and protein levels of proinflammatory cytokines in the hippocampal CA1 area of rats after cerebral ischemia. One week after four-vessel occlusion (4-VO) surgery, rats were intraperitoneally administered saline (4-VO), paeoniflorin (4-VO+PF; 10, 20, 40 mg/kg/d) or HU308 (4-VO+HU308; 3 mg/kg/d) for consecutive 28 days. (A) Rats were sacrificed after 28 days consecutive drug treatment. Representative photomicrographs of hematoxylin-eosin-stained hippocampal regions of rats are shown in different groups: (a, g) sham-operated group, (b, h) 4-VO-operated group, (c, i) 4-VO+10 mg/kg/d PF group, (d, j) 4-VO+PF+20 mg/kg/d group, (e, k) 4-VO+40 mg/kg/d group or (f, l) 4-VO+3 mg/kg/d HU308 group. Boxed regions in a–f are shown in j–l, respectively. Scale bar: 50 µm. (B) Neuronal cell density in CA1 region was measured. (C) The protein levels of proinflammatory cytokines including IL-1β, TNF-α and IL-6 in the hippocampal homogenate were measured by enzyme linked immunosorbent assay. [file 13020_2018_173_MOESM2_ESM.tif]

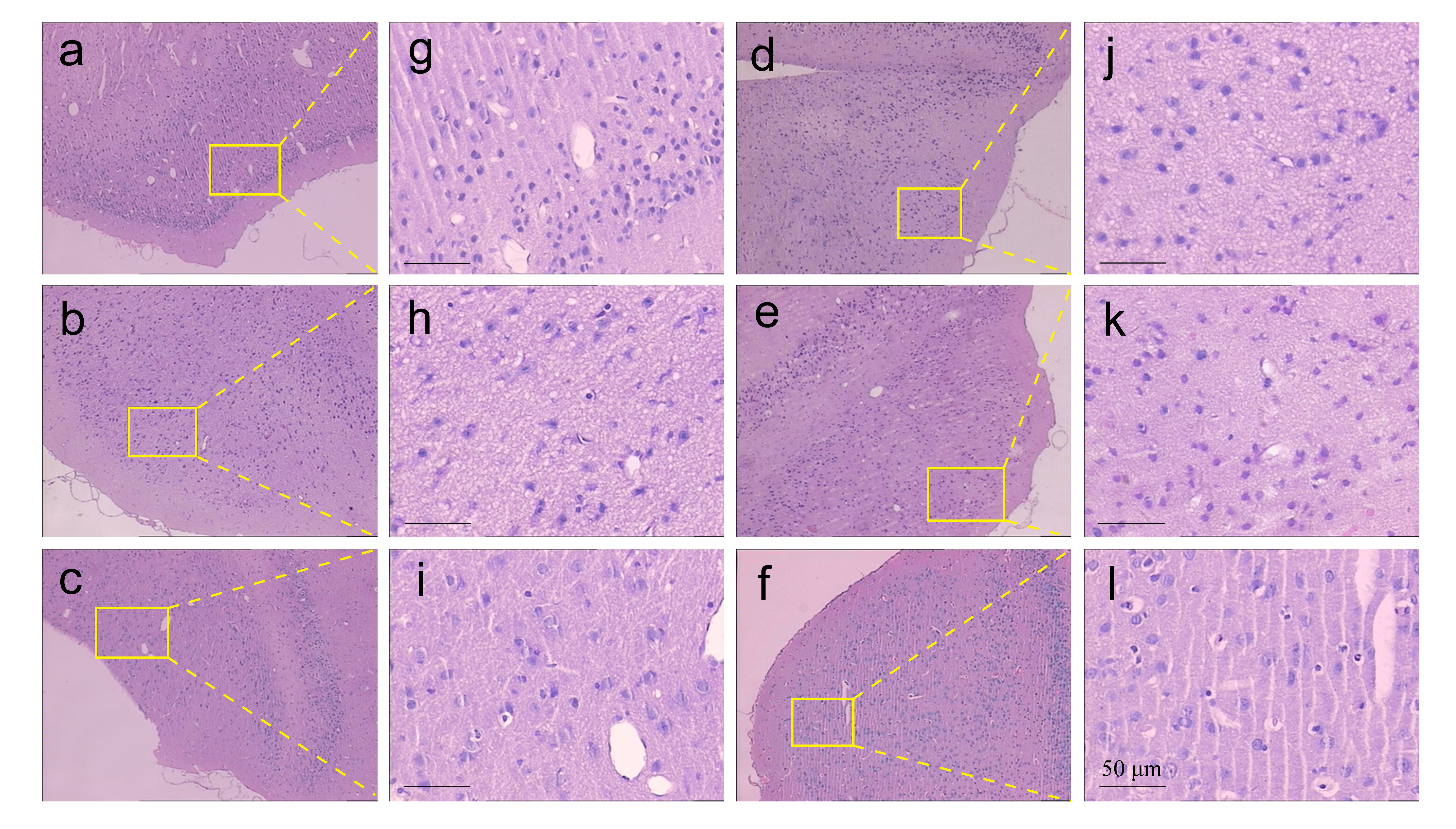

Supplement: Supplementary file 3 — Additional file 3: Figure S2. Effects of paeoniflorin on histopathology of the entorhinal cortex area of rats after cerebral ischemia. Representative photomicrographs of hematoxylin-eosin-stained entorhinal cortex region of either sham-operated rats (a, g) or rats that had been subjected to four-vessel occlusion followed by the treatment with saline (4-VO; b, h), paeoniflorin (4-VO+PF; 40 mg/kg/d; c, i), paeoniflorin+AM630 (4-VO+PF+AM630; 40 + 3 mg/kg/d; d, j), AM630 (4-VO+AM630; 3 mg/kg/d; e, k) or HU308 (4-VO+HU308; 3 mg/kg/d; f, l) for consecutive 28 days. Boxed regions in a–f are shown in j–l, respectively. Scale bar: 50 µm. [file 13020_2018_173_MOESM3_ESM.tif]

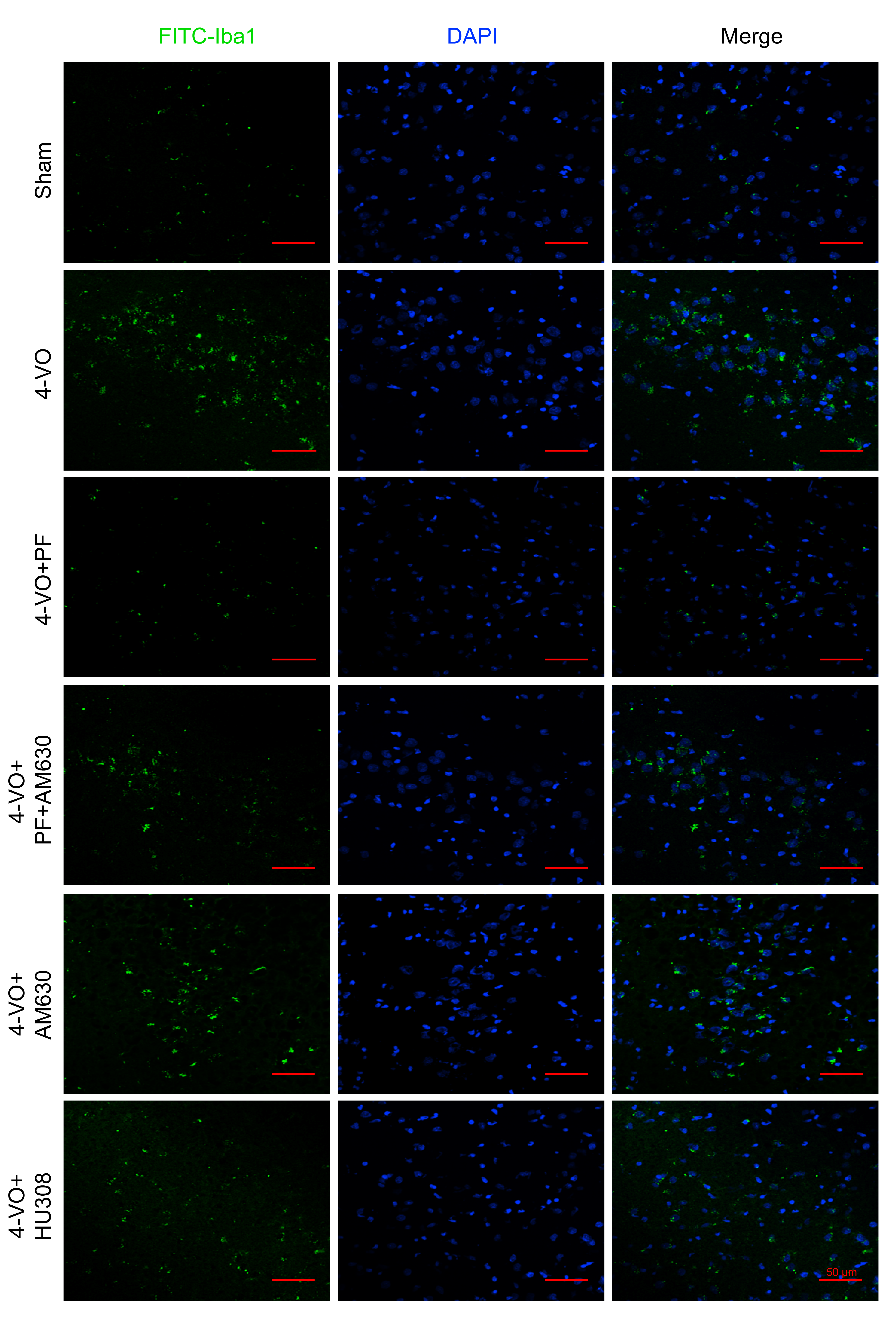

Supplement: Supplementary file 4 — Additional file 4: Figure S3. Effects of paeoniflorin on the activation of microglial cells in hippocampi of rats after cerebral ischemia. One week after four-vessel occlusion (4-VO) surgery, rats were intraperitoneally administered saline (4-VO), paeoniflorin (4-VO+PF; 40 mg/kg/d), paeoniflorin+AM630 (4-VO+PF+AM630; 40 + 3 mg/kg/d), AM630 (4-VO+AM630; 3 mg/kg/d) or HU308 (4-VO+HU308; 3 mg/kg/d) for consecutive 28 days. After treatment, hippocampus tissue sections were co-stained for Iba1 (an activated microglia marker; green) or DAPI (blue). The images were observed and captured by a confocal laser scanning microscope. Scale bar: 50 μm. [file 13020_2018_173_MOESM4_ESM.tif]

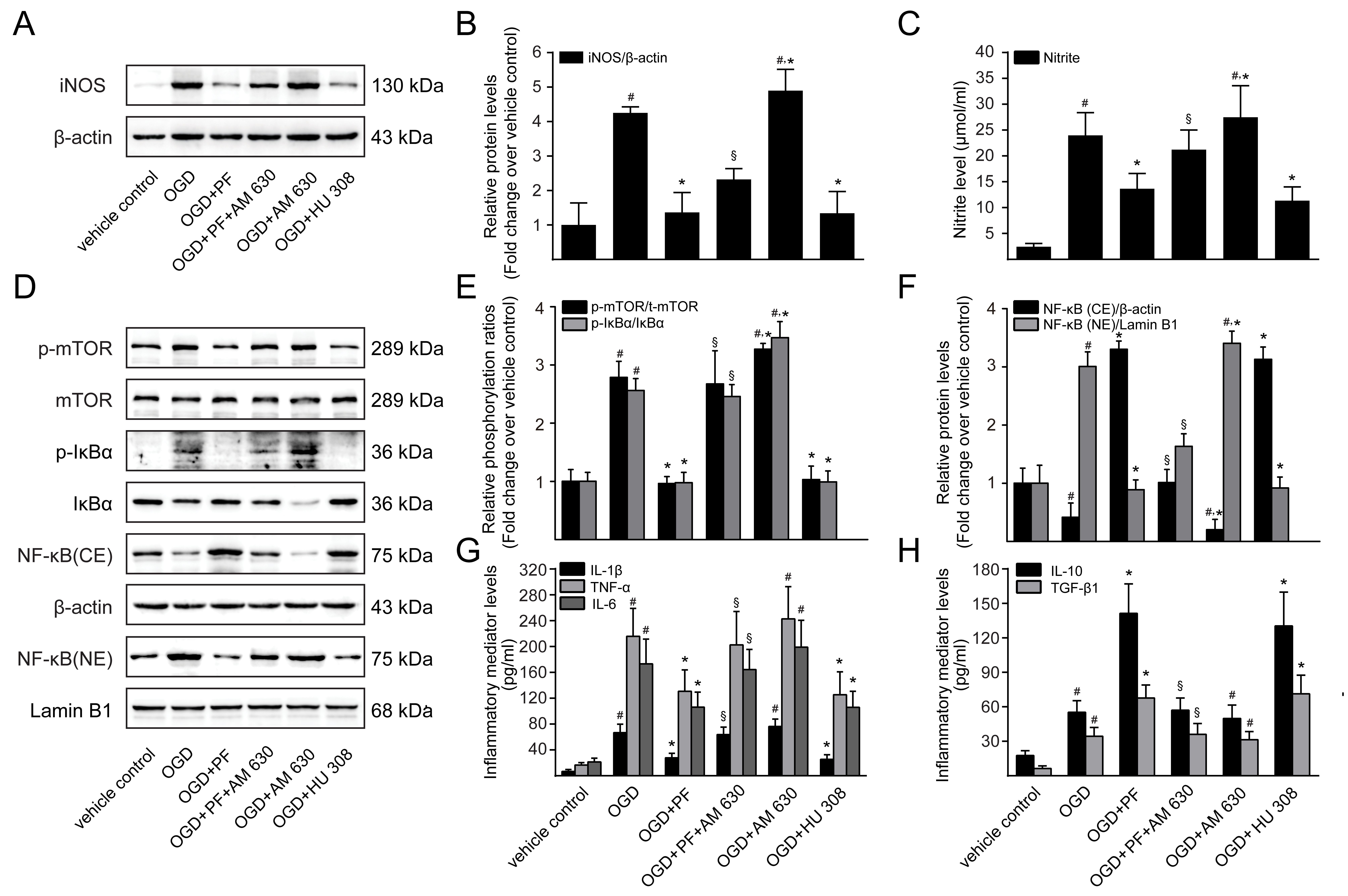

Supplement: Supplementary file 5 — Additional file 5: Figure S4. Effects of PF on M1/M2 polarization and mTOR/NF-κB signaling pathway in BV-2 microglia exposed to oxygen glucose deprivation (OGD). BV-2 cells were pre-incubated with DMSO (vehicle), PF (50 μM), AM630 (2 μM) or HU308 (10 μM) for 4 h followed by OGD for 6 or 24 h. (A) The cell lysates were immunoblotted with iNOS or β-actin antibody. β-Actin served as a loading control. These results are representative for three independent experiments. (B) The differences of the protein expression between the groups were analyzed with Image J. (C) The levels of nitrite in cell culture supernatants were determined by the Griess reaction. (D) Representative Western blotting photographs showing protein levels of p-mTOR, mTOR, p-IκBα, IκBα, NF-κB and β-actin in cytoplasmic fractions and NF-κB and lamin B1 in nuclear fractions of BV-2 microglia. The β-actin and lamin B1 protein levels were used as internal controls, respectively, for cytoplasmic extracts (CE) and nuclear extracts (NE). (E, F) The protein levels of phosphorylated and total mTOR and IκBα in cytoplasmic extracts and NF-κB in cytoplasmic and nuclear extracts, were converted to arbitrary densitometric units, normalized by the value of the corresponding loading controls and expressed relative to the phosphorylation ratio or to the protein levels in vechile control (defined as 1-fold). (G, H) The levels of IL-1β, IL-6, TNF-α, IL-10 and TGF-β1 in cell culture supernatants were determined by enzyme-linked immunosorbent assay. Each bar represents mean ± SD of three independent experiments. #P < 0.05 versus vehicle control group, *P < 0.05 versus OGD group, §P < 0.05 versus OGD+PF group. [file 13020_2018_173_MOESM5_ESM.tif]
